# User-centred digital solution for dentofacial deformities management: an innovative approach technology based

**DOI:** 10.3389/fdgth.2025.1502853

**Published:** 2025-03-06

**Authors:** Vincenzo Abbate, Emanuele Carraturo, Giulia Togo, Stefania Troise, Federica Calabrìa, Mauro Cataldi, Daniela Adamo, Guido Iaccarino, Maddalena Illario, Riccardo Nocini, Giovanni Dell'Aversana Orabona

**Affiliations:** ^1^ Department of Neurosciences, Reproductive and Odontostomatological Sciences, University of Naples Federico II, Naples, Italy; ^2^ Department of Clinical Medicine and Surgery, University of Naples Federico II, Naples, Italy; ^3^ Department of Public Health, University of Naples Federico II, Naples, Italy; ^4^ Otolaryngology Department, University of Verona, Verona, Italy

**Keywords:** full digital approach, Blueprint, dentofacial deformities, computer assisted surgery, communication technology

## Abstract

**Objective:**

Dentofacial deformities (DFD) encompass a range of conditions affecting approximately 60% of the population, varying from mild to severe cases. Managing these disorders requires a multidisciplinary approach, yet establishing a unified therapeutic protocol across different specialties often proves challenging. This underscores the necessity to understand the specific needs and demands of patients diagnosed with DFD, aiming to develop an effective treatment pathway. The objective of our study is to identify the unmet needs of patients with DFD and to propose digital solutions, based on Information and Communication Technology (ICT), that can help patients meet these needs by improving the diagnostic-therapeutic pathway.

**Methods:**

By examining the medical records of 147 patients diagnosed with DFD, a team of specialists created the profile of a hypothetical DFD patient, termed “Persona” by using the “Blueprint- persona”. This Persona theoretically represents the broader DFD patient population, capturing their needs, demands, goals, problems, and challenges. Based on these findings, a comprehensive ‘DFD Management Pathway’ is proposed, encompassing both general and specialized preoperative and postoperative consultations required for these patients.

**Results:**

The identified unmet needs of a typical DFD patient were psychological support, nutritional support and diet recommendations, advice on oral hygiene, assistance by specialized professionals. The proposed digital solutions were the use of video tutorials and online courses, daily notifications on applications on smartphones, social media channel and multidisciplinary platform.

**Conclusion:**

This study highlights that the Blueprint methodology proves instrumental in pinpointing specific characteristics and unmet needs of various patient groups. The critical gaps in the diagnostic-therapeutic pathway for patients with dentofacial deformities and underscores the potential of digital solutions in addressing these unmet needs. By creating a detailed patient Persona, by using The Blueprint methodology, and mapping their challenges, psychological support, nutritional guidance, oral hygiene advice have been identified as unmeet needs. The proposed digital tools, including video tutorials, online courses, smartphone notifications, social media channels, and multidisciplinary platforms, provide a promising avenue to enhance patient engagement, streamline care delivery, and improve overall treatment outcomes. Future research should focus on validating these solutions in clinical settings to ensure their feasibility and effectiveness in addressing the unique demands of DFD patients.

## 1 Introduction

The umbrella term *Dentofacial deformities* (DFD) identifies a group of dental and skeletal alterations that result in alterations in the relationship between the jaws, causing unattractive facial features and occlusal impairment (1, 2). Research indicates that 35% of the population has normocclusion, 60% suffer from moderate to severe malocclusion, and 5% have severe dentofacial deformities, including cases in syndromic patients. In particular, 75% of children between 6 and 12 years old and 88% of teenagers between 12 and 17 years old have a malocclusion. This means that it is a worsening problem, which does not improve with the passing of time and the growth (3). But malocclusion should not be considered only a dental or aesthetic problem, as a general problem that affects several features. In fact DFD is a complex disease, considering respiratory, eating and psychological disorders (4–6). Issues such as obstructive sleep apnea are frequently linked to dento-skeletal malocclusion, which narrows the upper airway excessively. A retracted chin position, for example, can restrict air passage due to this malocclusion. Therefore, managing the upper airways has become a crucial aspect of bimaxillary repositioning surgery (7, 8). Another important aspect to consider is feeding, because these patients frequently present eating disorders (9). Moreover, this condition significantly impacts the quality of life, due to the patients' perception to be less attractive, and, consequently, to have lower chances of being socially acceptable and to live a successful life (3–5). Thus, although surgical treatment is the mainstay of DFD treatment, a multidisciplinary approach involving several health professionals, maxillofacial surgeon, orthodontist, nutritionist, psychologist/psychiatrist, speech therapist, is required to manage this complex pathology (5–7).

While Computer-aided design (CAD) and Computer-aided manufacturing (CAM) technologies have revolutionized DFD surgical planning and treatment (10–13), computer technologies still have a minor role in the diagnosis and management of the complex symptoms that accompany facial deformities or complicate their surgical treatment, such as psychological or nutritional problems. This can be due to the lack of a standardized and individualized multi-specialistic care pathway to optimize the comprehensive management of the unmet needs of DFD patients both pre- and post-operatively (13, 14). Nonetheless, over the past two decades, Information and Communication Technology (ICT), including artificial intelligence, mHealth, and telemedicine, has steadily gained importance to address similar issues in routine clinical practice. These technologies provide innovative communication methods and efficient organizational strategies (15). To facilitate the implementation of these ICT interventions also though a detailed identification of patients' needs, in recent years, the Blueprint “Personas” methodology has been developed as a tool for promoting person-centered care. This methodology identifies typical profiles of patients affected with specific diseases, which include information about socio-economic status, personal, health and environment (home setting) needs, and computer literacy so that digital technologies can be implemented and leveraged to obtain personalized care pathways. This methodology has been successfully adopted in other healthcare contexts, such for metabolic syndrome, obstructive apnea syndrome and pulmonary diseases, burning mouth syndrome and cardiovascular congenital defects (16, 17). Starting from these considerations, the objective of our study is to identify the unmet needs of patients with DFD by using the Blueprint persona approach, and to propose digital solutions, based on Information and Communication Technology (ICT), that can help patients meet these needs by improving the diagnostic-therapeutic pathway.

## 2 Materials and methods

### 2.1 Study design

Within the DHEAL-COM project, a retrospective review on clinical database and phone survey was administered to 147 patients treated at Maxillofacial Unit in Federico II University Hospital for Dentofacial Deformities, from 01 September 2017 to 31 September 2023, to analyze the demographic, clinical and social characteristics. Twelve patients were excluded from the final cohort since they either did not give consent for personal data processing, or it was not possible to infer general epidemiological characteristics from medical records.

### 2.2 Blueprint methodology

This study utilized the European Commission's “Blueprint on Digital Transformation in Health and Care in an Aging Society” (Blueprint) methodology. This approach is designed to identify and specify key digital solutions and high-impact user scenarios, specifically tailored to the needs of DFD patients treated at the Maxillofacial Unit of Federico II University Hospital in Naples, Italy (European Commission, 2023). The Blueprint “Personas” methodology was instrumental in this process, enabling the detailed design and identification of patients' unmet needs. This methodology has been successfully applied in various medical fields previously (16, 17). The Blueprint persona serves as a tool for advancing person-centered care by outlining patient profiles that encompass a range of factors, such as individual characteristics, socio-economic background, health status, and environmental influences, particularly within the home environment. It also highlights the possible advantages of utilizing digital resources for the patient and other key individuals involved in their care, including caregivers (both informal and professional), healthcare workers, service providers, and researchers. The Blueprint, a pillar of the European Innovation Partnership on Active and Healthy Ageing (EIP-AHA), focuses on active and healthy ageing by improving health services, promoting the sustainability of systems and stimulating economic growth through the Silver Economy. At the core of its strategy is a dynamic matrix that categorises 12 “personas” according to four life stages and three health conditions. This tool helps healthcare professionals address the complex and diverse needs of older people. Each person represents a patient archetype, with specific characteristics, behavioural traits and evolving health needs. This methodology has been successfully applied to conditions such as Burning Mouth Syndrome (BMS) and temporomandibular disorders (TMD) (18). By integrating digital technologies and customising solutions according to the specific needs of patients, the Blueprint promotes independence, improved quality of life and active economic participation of patients. In our study, the blueprint methodology was useful to condense into a single person, all the unmet needs of patients with dentofacial deformities. Therefore, the Blueprint can be imagined as the digital twin of a patient with DFD.

### 2.3 Identification of DFD's profile

The Blueprint methodology is based on an interdisciplinary Focus Group, that through the analysis of patients' medical records and through the evaluation of specific questionnaires submitted to patients, is able to identify the DFD patient's digital twin. This approach condenses all the characteristics and needs of a specific category of patients into a single digital person. The Focus group comprised 2 surgeons, 2 orthodontists, 2 nutritionists, 1 speech therapist, 1 psychologist, and 1 pharmacologist. They gathered patient information through telephone interviews and clinical reports, including age, sex, gender, and occupation. Using this data, the Focus Group identified the typical profile of a DFD patient, referred to as “Persona”. This hypothetical, specific patient represents a broader patient population (World Health Organization, 2019) (16, 17). The “Persona” was given a realistic name and used as a digital twin to describe the needs, demands, goals, problems, and difficulties of this group. Behavioral characteristics were also considered for their potential impact on the outcomes of proposed interventions, both short-term and long-term.

The study employed the European Commission's “Blueprint on Digital Transformation in Health and Care in an Aging Society” methodology (19, 20). To identify unmet needs, patients were interviewed with standardized questionnaires made by the focus group. The questions covered 3 topics:

– Topic I: Tasks and goals to be achieved to support the target population. Defining the content of the intervention;
– Topic II: Evaluation of technical alternatives to be included in the intervention;
– Topic III: Strategies to reduce the consequences of limited health literacy (Table 1).

**Table 1:** Questionnaire administrated to the patients.

Topic 1: Tasks and goals to be achieved to support the target population. Defining the content of the intervention
Question 1: What goals do you think should be achieved to support the needs of the target population?
“What are the most felt needs of the target population?”
“What daily and social activities do you think are most compromised by the disease?”
“Have you ever proposed/used a technological device to support self-management of health status?”
“What was the worst experience you had as a patient?”
“What are the heaviest daily challenges for you and how do you deal with them?”
“What have you learned that can also be valid for similar situations?”
Question 2: What content would you like to convey through intervention with technology?
“What do you think is the missing content that we should introduce to support you?”
Topic 2: Evaluation of technical alternatives to be included in the intervention
Question 3: Looking at the proposed technologies, what is your impression? How can we make them more effective in supporting the target group?
“Do you think the proposed technologies are usable?”
“Who is the ideal user for this technology in your opinion?”
“What is your preferred solution?”
“What obstacles do you see in the use of these technologies?”
“Try to imagine using this technology in your daily life: do you think it is easy to introduce it into your home?”
“Do you think you need help using these devices or training?”
Topic 3: Strategies to reduce the consequences of limited health literacy
Question 4: What do you think is essential to include in a training course to help patients with limited health literacy and eHealth?
“What kind of topics do you want to address in the training?”
“What kinds of problems do you encounter in practice that you would like to see addressed in the training?”
Question 5: What are the most effective ways of learning to engage the patient that you have used in your clinical experience?
“Do you suggest apps or technologies to support clinical prescribing?”
“What strategies do you use to inform yourself about the use of a technological device for your health?”
“What difficulties have you encountered when learning how to use technology for your health?”
“When do you think it is important to introduce eHealt and Healt Literacy training?” Who is the ideal “student “in your opinion?”
Question 6: What should this formation look like?
“What do you think is the best way to deliver the training?”
“Among the various stages of the disease, when would it be best to do an information/education intervention?”

From interview results and medical records, a theoretical elaboration of the Blueprint persona was created. Healthcare professionals, along with experts in Digital Health, discussed and identified four main technological macro-areas, an imaging protocol, and the necessary professional roles to establish a diagnostic workflow. This workflow aims to address the unmet needs of DFD patients effectively. The team then translated these findings into applicable digital health solutions.

## 3 Results

### 3.1 Population

Of the 135 patients included in the study, the majority were female (67%). The age distribution was as follows:

• 33% of patients were between 18 and 25 years old.
• 49% were between 26 and 40 years old.
• 18% were over 40 years old.

Regarding the clinical presentation, the most common diagnosis was Class III malocclusion (62%), followed by Class II malocclusion (28%) and facial asymmetry (10%). All the patients underwent presurgical orthodontic treatment and orthognathic surgery to correct the dentofacial deformities. The questionnaires were submitted to all the patients and a notable finding was that patients with Class III malocclusion were more likely to report psychological distress and lower quality-of-life scores compared to those with Class II malocclusion, highlighting the psychosocial impact of this condition.

### 3.2 Persona

Drawing from the identified “Persona” for DFD patients, the Focus Group established a therapeutic protocol and outlined the necessary professional roles to address the specific needs of these patients. A detailed Blueprint “Persona”, named Mariangela, was created to embody the characteristics, recurring issues, and needs highlighted through the survey. Mariangela, a 37-year-old woman residing in the suburban areas of Naples, is a young, unmarried lawyer without children. Diagnosed with a class III dentoskeletal malocclusion at the age of 11, this congenital condition progressively worsened over time, causing both functional and aesthetic complications. Mariangela experienced frequent headaches, particularly upon awakening, and was reported to have snoring and apnea episodes during sleep. This led to chronic fatigue and relationship challenges due to her disharmonious appearance.

The Focus Group underscored that complex cases like Mariangela could greatly benefit from custom-designed mHealth (mobile health) solutions tailored to their specific needs. Mariangela required psychological support to address depression and an eating disorder. She also needed a dedicated figure or technological support to guide her through the orthodontic-surgical process. Additionally, a therapeutic protocol was necessary to improve her aesthetic appearance, dental occlusion, and sleep apnea episodes. A summary of Mariangela's baseline situation is shown in Table 2.

**Table 2:** Blueprint “persona” data set.

Persona use case	Health domain	Data	Tools	Settings of the service provision	Interoperability requirements
Mariangela- DFD	Nutrition	Diet, Oral Hygiene	Video tutorial, Massive Open Online Courses (MOOCs)	Community, hospital, patient home	GPs software, Hospital software, mobile tools
	Psycologist assistance	QoL tests	Online multidisciplinary platform	Community, hospital, patient home	GPs software, Hospital software, mobile tools
	Clinical data (respirator)	AHI, saturation	Polysomnograph, Rhinomanometer through telemonitoring	Community, hospital, patient home	GPs software, Hospital software, mobile tools
	Psychological assistance	Peers comunication	Dedicated social media	Patient home	Patient software, mobile tools
	Dentofacial Deformity (Preoperative diagnosis)	CT Cone Beam imaging, Denture scans, 3D facial scans	CBCT scan, intraoral scan device, facial scanner	Hospital	Hospital software, hospital equipment

### 3.3 ICT technology

The analysis of the unmet needs of patients with DFD shows a lack of home telematic communication and a sense of abandonment on the part of a particular type of patient who needs constant remote support and updating. Therefore, the identification of digital solutions for remote support is fundamental. Following this consideration, four primary technological macro-areas were identified to fulfill their unmet requirements:

1. **Health Information Exchange**: Implementation of secure and reliable portals for health information search to facilitate access to relevant medical data and supporting communication between multidisciplinary teams of health care professionals and patients.
2. **Tele-Monitoring**: Utilization of electronic diaries featuring visual and auditory reminders, coupled with the assessment of health parameters to monitor patient progress remotely.
3. **Education, Health, and Digital Health Literacy**: Promotion of healthy cooking and eating habits, along with Massive Open Online Courses (MOOCs) to educate patients on their conditions and treatments.
4. **Social or Peer Support**: Establishment of social networks and messaging platforms to maintain continuous communication between caregivers and patients.
5. **Digital planning**: Generate the patient's “digital twin” to be able to plan therapeutic procedures remotely to reduce hospital admissions.

Additionally, a comprehensive imaging protocol was delineated for accurate pre-surgical evaluation, comprising:

• TC Cone Beam imaging
• Denture scans
• 3D facial scans
• Psychological assessments
• Rhinomanometry
• Polysomnography

An interdisciplinary team, encompassing the following professional roles, was identified to provide the necessary support for DFD patients:

• Maxillofacial Surgeon
• Orthodontist
• Psychologist
• Speech Therapist
• Nutritionist
• Pharmacologist

These measures aim to ensure a holistic and effective management approach for patients with DFD, addressing their diverse and specific needs through integrated technological solutions and expert care (Table 3).

**Table 3:** Information communication technology & tools to meet the unmet needs.

Unmet Need	Information Communication Technology ICT	Outcomes	Setting	Tools
Assistance with feeding	Education, Health, and Digital Health Literacy Promotion of healthy cooking and eating habits, along with Massive Open Online Courses (MOOCs) to educate patients on their conditions and treatments	Assessing the improvement of the eating disorder through psychological testing. Assessing BMI during the course of treatment	Patient home	Video Tutorial
Assistance in managing oral hygiene				Video Tutorial
Dietary recommendations				Massive Open Online Courses (MOOCs)
Psychological assistance	Health Information Exchange Implementation of secure and reliable portals for health information search to facilitate access to relevant medical data and supporting communication between multidisciplinary teams of health care professionals and patients	Assess through specific questionnaires the degree of resolution of the disorder. improve aesthetic dispersive disorder	Patient home	Online Multidisciplinary platform
Facilitated communication with care professionals involved				
Need to exchange opinions with other DFD patients	Social or Peer Support Establishment of social networks and messaging platforms to maintain continuous communication between caregivers and patients	Support communication between patient and care professional. Reducing hospital admissions.	Patient home	Social media channel dedicated to DFD
Psychological assistance				
Breathing difficulties during sleep	Telemonitoring Utilization of electronic diaries featuring visual and auditory reminders, coupled with the assessment of health parameters to monitor patient progress remotely	Reduce the number of sleep apneas (AHI index) Improve sleep quality and work performance	Hospital, Patient home	Polysomnograph Rhinomanometer
Treating dental malocclusion	Digital planning Generate the patient's “digital twin” to be able to plan therapeutic procedures remotely	Reducing hospital admissions.	Hospital	Optical sensors for 3D scanning Operational tools

### 3.4 DFD management pathway

**Figure 1:**
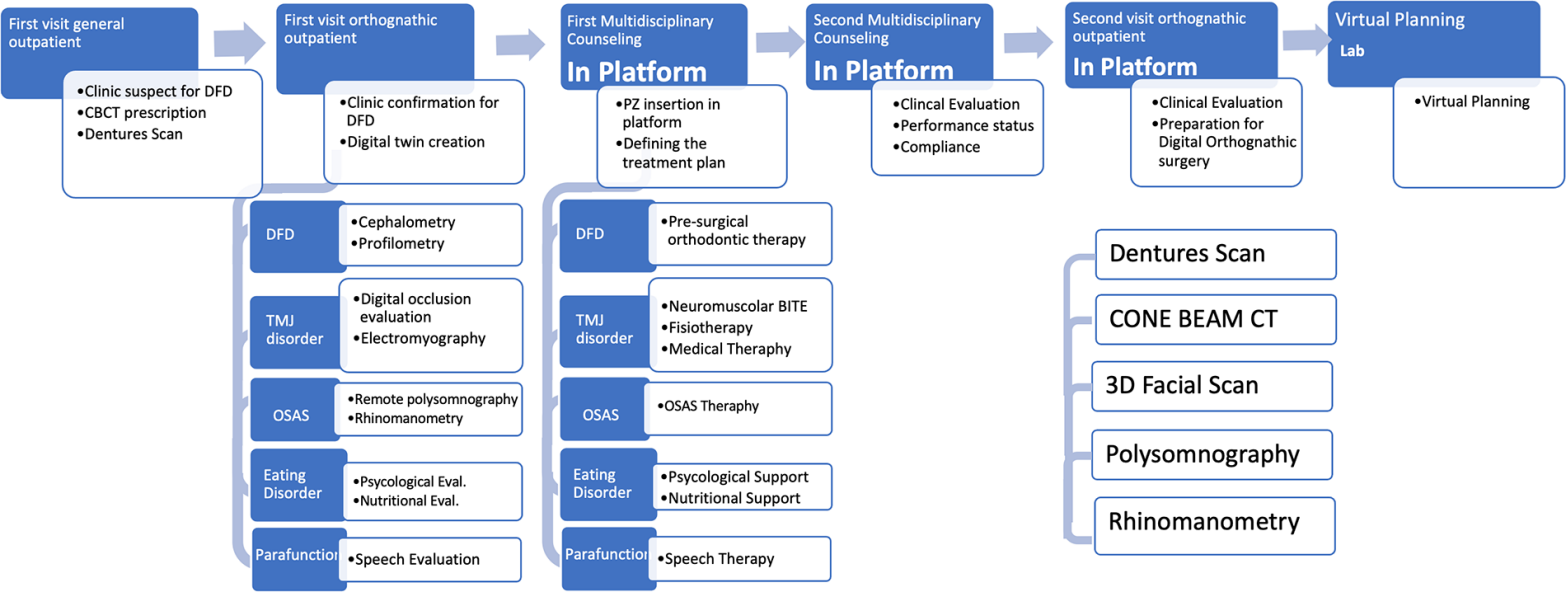
Preoperative diagnostic and therapeutic pathway.

**Figure 2:**
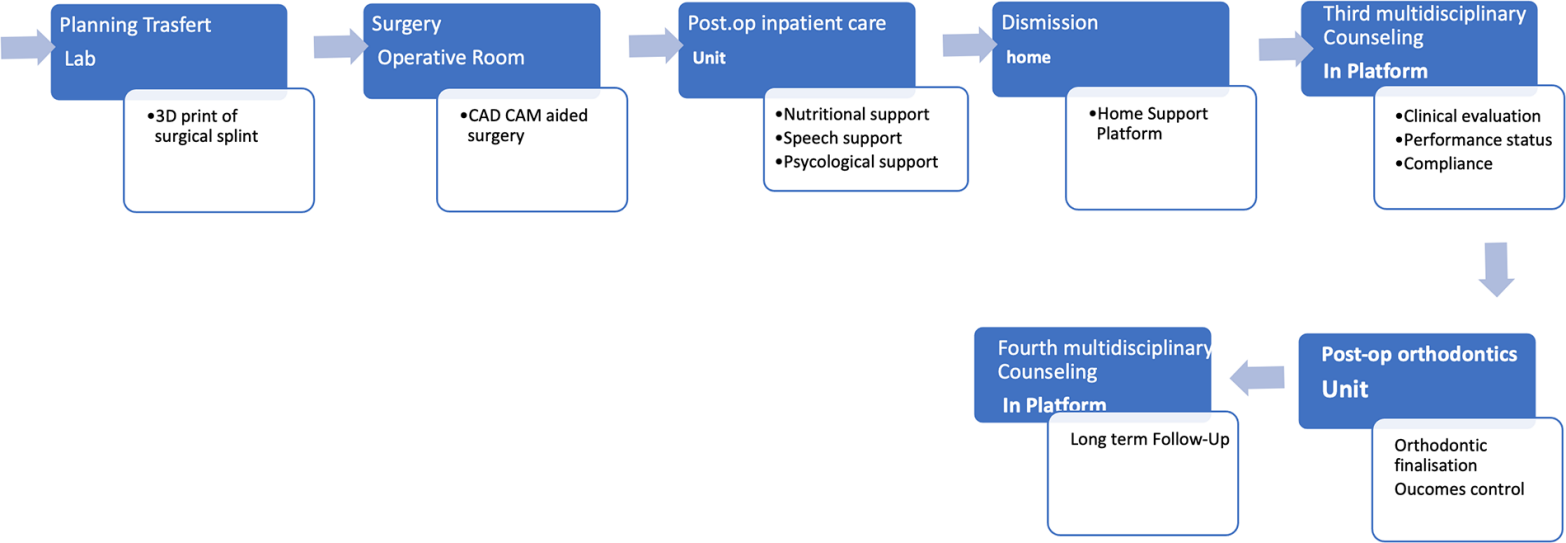
Postoperative diagnostic and therapeutic pathway.

Based on these findings, a detailed “DFD Management Pathway” has been established. This pathway encompasses a full spectrum of care, including all necessary general and specialized visits, as well as clinical and instrumental investigations required for effective management. It covers both preoperative and postoperative stages, ensuring a thorough approach to address the needs of DFD patients. The entire pathway, with its various components and stages, is concisely summarized in Figures 1, 2.

## 4 Discussion

A nice facial appearance depends on the harmony of craniofacial components. Any growth phase alterations can lead to dento-skeletal or dento-facial deformities, characterized by improper relationships between teeth and supporting structures. These issues cause functional and aesthetic problems, with psychological impacts, especially in adolescents. Research shows that 35% of the population has normocclusion, 60% has moderate to severe malocclusion, and 5% has severe deformities, including syndromic cases. Normally, the tongue and muscles create balanced forces between the dental arches, but malocclusion disrupts this balance. Attention is growing on respiratory dynamics in patients with dentofacial deformities, as conditions like obstructive sleep apnea often correlate with malocclusion, which narrows the airway (8, 21–23). Managing the upper airways is key in bimaxillary repositioning surgery. Corrective surgery aims to reposition the maxillary bases, restore occlusion, improve masticatory function, harmonize facial proportions, ensure stability, and achieve patient satisfaction (24). Over time, the diagnosis and surgical planning for dentofacial deformities have evolved from 2D methods like orthopantomography and plaster models to 3D virtual planning using CT, CBCT, intraoral scans, and facial scans, which allows for more precise, efficient, and patient-friendly surgery (25–27).

While technological advancements have increased predictability, efficacy, and simplicity in the surgical phase, there remains a lack of unified treatment protocols across the entire care pathway, considering the various professionals involved and the clinical and instrumental investigations required (27–31). Thus, the necessity to assess and develop tools to meet the needs of typical orthognathic patients and identify their unmet needs. The Blueprint “Personas” approach, formulated by the “Blueprint on Digital Transformation in Health and Care in an Ageing Society”, offers an intuitive process for identifying specific patient subsets' unmet needs (World Health Organization 2019) (16–19, 32). This study aims to identify key digital solutions and user scenarios for individuals with a dentofacial deformity profile, adapting the “Blueprint” user-centered design methodology. As part of the DHEAL-COM project, a phone survey (Table 1) was conducted with 135 patients treated at the Maxillofacial Unit of Federico II University Hospital from 2017 to 2023, to analyze their demographic, clinical, and social characteristics. An interdisciplinary Focus Group developed the theoretical “Dentofacial deformity persona” (Mariangela). Analyzing data from 135 patients and identifying frequently occurring characteristics (gender, age, education, comorbidities).

A number of unmet needs were highlighted for these patients (Table 2). These include the need for psychological support throughout the pre- and post-operative pathway. As highlighted by Dons et al. (33) up to 13% of BDDs have disorders related to self-image dissipation requiring targeted behavioral treatment, so-called Body Dysmorphic Disorder (BDD).

Body Dysmorphic Disorder (BDD) is a mental health condition characterized by an obsessive focus on one or more perceived flaws in physical appearance, which are either minor or not observable to others. Individuals with BDD can spend hours a day thinking about their perceived defects, leading to significant distress and impairments in daily functioning. In DFD patients this disorder is definitely more highlighted (33). Furthermore, Madhan et al. (34) showed that patients with DFD have a higher prevalence of Temporo Mandibular Disorders TMD (14 to 97%) and oro-facial pain (33%) compared to those without. This condition aggravates the patient's symptom picture with pain and chewing disorders that need proper framing as early as preoperatively (34). Also fundamental for these patients is a speech therapist support: as described by Farronato et al. (35) there are significant correlations between malocclusion and dyslalia. The presence of Class III occlusion, diastema, increase in overjet, presence of open and deep bite, asymmetry have high tendency to be associated with speech disorders such as dyslalia. Another unmet need pertains to the need for a common platform for dialogue with the various professionals on the multidisciplinary team. The Nutritional management for these patients should also not be neglected for two main reasons: firstly, since the surgery directly affects the oral apparatus and chewing mechanisms, controlled nutrition, and the use of liquid or semi-liquid diets are essential, especially in the initial weeks post-surgery (9). Secondly, considering caloric intake and the distribution of macro- and micro-nutrients is vital; patients require a complete, possibly high-calorie diet for proper healing of bone fractures (36, 37). Last but not least, as firstly demonstrated by Miyao et al. malocclusion, specifically overjet, was associated with the severity of OSAS in non-obese patients, suggesting that malocclusion may play an important role in the development of sleep apnea/hypopnea (38).

The blue print methods enabled easy and accurate identification of ICT needed to satisfy unmet needs. Five key technological areas were pinpointed to address these patients' unmet needs:

1. Health information exchange;
2. Tele-monitoring;
3. Education, including gamification and health/digital health literacy, empowerment;
4. Social or peer support/social networks, messaging.
5. Digital planning

Based on these insights, a common care pathway incorporating key digital solutions was defined. This pathway (Figures 1, 2) was structured around pre- and postoperative management: in the preoperative phase, a set of clinical and instrumental investigations and necessary specialist outpatient visits were standardized. The key points are: appropriate preoperative imaging (CT cone beam, intraoral scans, cephalometry), proper framing of airway disorders with rhinomanometry and polysomnography, nutritional and speech evaluation for setting an adequate diet and psychological support.

The postoperative pathway standardized the order of specialist outpatient visits, enabling patient support throughout the healing process and their acceptance of their new image, both through in-person visits and remotely via the platform (key digital solution). The pathway includes a total of 2 in-person outpatient visits aimed at giving an initial framing of dentoskeletal dysmorphia and defining the correct diagnostic-therapeutic pathway and includes 4 platform meetings (telemedicine). The latter have the function of reducing in-person visits and improving interdisciplinary communication between the specialist figures involved in the management of the pathology. They also have the benefit, by avoiding excessive physical travel, of improving patient compliance with treatment, thus being able to bring patients from distant geographic realities closer to the proposed therapeutic pathway and thus favoring the phenomenon of “health migration”.

Certainly, our work is a preliminary study and has limitations, first the lack of validation of the realized pathway; our future perspective is to put the pathway into practice and validate it through an implementation protocol. Other limitations regard: technology literacy among the patients and healthcare providers; issues related to multidisciplinary coordination. Although the study has these limitations, the most important impact concerns health equity, considering that by exploiting these digital solutions even patients in remote and patients by underserved areas can benefit from these services.

## 5 Conclusions

This study highlights that the Blueprint methodology proves instrumental in pinpointing specific characteristics and unmet needs of various patient groups. In fact, this approach is simply based on creating a digital twin of a specific category of patients, condensing all the characteristics and needs of the different patients of that category, in order to search for solutions to satisfy these unmet needs more quickly. By creating a detailed patient Persona, by using the Blueprint methodology, unmet needs of DFD patients have been identified, such as psychological support, nutritional guidance, oral hygiene advice. The critical gaps in the diagnostic-therapeutic pathway for patients with dentofacial deformities underscores the potential of digital solutions in addressing these unmet needs. The proposed digital tools, including video tutorials, online courses, smartphone notifications, social media channels, and multidisciplinary platforms, provide a promising avenue to enhance patient engagement, streamline care delivery, and improve overall treatment outcomes. Future research should focus on validating these solutions in clinical settings to ensure their feasibility and effectiveness in addressing the unique demands of DFD patients. Once the real-world validation of the model and of the questionnaire has been obtained, this methodology could be used as a model for others complex health condition requiring multidisciplinary care.

## Data Availability

The raw data supporting the conclusions of this article will be made available by the authors, without undue reservation.
